# Case Report: Early management and successful thrombolysis in two cases of high-risk pulmonary embolism

**DOI:** 10.3389/fmed.2026.1715383

**Published:** 2026-05-26

**Authors:** Yan Zhang, Jie Li, Yuquan Deng, Jinfeng Meng, Wei Zhou, Faxin Luo, Wangsheng Deng

**Affiliations:** Department of Emergency Medicine, People's Hospital of Longhua, Shenzhen, China

**Keywords:** cardiac arrest, pulmonary embolism, resuscitation, thrombolysis, urokinase

## Abstract

Through analyzing two cases of acute fatal pulmonary embolism, we emphasize the critical importance of early diagnosis, timely risk assessment, and precise thrombolytic therapy, particularly for hemodynamically unstable patients. Key aspects such as timing, dosage, and multidisciplinary management are discussed to optimize patient outcomes.

## 1 Introduction

Pulmonary embolism (PE) is a common non-cardiac cause of sudden cardiac arrest, with acute fatal cases accounting for a substantial proportion of such events (1). According to the 2022 European Society of Cardiology (ESC) guidelines, high-risk PE is defined by hemodynamic instability, including sustained hypotension, shock, or cardiac arrest. Its symptoms are often nonspecific, making timely diagnosis challenging. The clinical manifestations of PE are diverse and frequently ambiguous, further complicating its identification (2). Sudden cardiac arrest occurs in approximately 90% of cases within 2 h of symptom onset, with high mortality rates if left untreated (3). Managing these cases requires a rapid multidisciplinary approach encompassing accurate diagnosis, immediate resuscitation, and evidence-based treatment strategies (4). This report highlights two successfully treated cases of high-risk PE at our hospital recently, focusing on early management and thrombolytic protocols that facilitated recovery. These cases are valuable as they illustrate successful use of immediate thrombolysis and intensive care coordination in cardiac arrest due to PE—an area with limited guideline consensus.

## 2 Case introduction

### 2.1 Case 1

A 51-year-old female was brought to our Emergency Department by her friends due to acute respiratory distress following 2 weeks of coughing. Initial assessment revealed severe hypoxemia (oxygen saturation 78%), cyanosis, delirium, hemodynamic collapse, tachycardia (136 bpm), and hypothermia (35 °C). Shortly after arrival, she suffered cardiac arrest; immediate cardiopulmonary resuscitation restored spontaneous circulation, but severe instability persisted, necessitating mechanical ventilation, intensive vasopressor and inotropic support, and cooling measures. Laboratory tests showed profound acidosis, critical hypoxemia, hypercapnia, elevated BNP (22,692 pg./mL), and increased D-dimer (2.21 μg/mL). A point-of-care focused echocardiogram, performed by the emergency medicine chief resident in the resuscitation bay, demonstrated findings consistent with acute massive pulmonary embolism and right ventricular strain. Given the persistent hemodynamic instability despite aggressive vasopressor support (norepinephrine 0.5 μg/kg/min) and the absence of contraindications to thrombolysis (no recent surgery, trauma, or bleeding history), the decision was made to proceed with intravenous thrombolysis. Bedside thrombolysis was immediately administered as follows: heparin sodium 3,000 IU intravenous bolus, followed by urokinase 400,000 IU diluted in 10 mL of 0.9% normal saline injected intravenously over 3 min, and then urokinase 600,000 IU diluted in 90 mL of 0.9% normal saline infused intravenously over 30 min (total urokinase dose 1,000,000 IU). The patient’s vital signs gradually stabilized thereafter. This intervention enabled subsequent successful pulmonary embolectomy. The patient improved steadily, regained consciousness by day four, and was discharged neurologically intact after 2 weeks.

### 2.2 Case 2

A 61-year-old female presented with acute respiratory distress and severe hypoxemia (SpO₂ 69%) after prolonged immobility during travel. Initial findings included agitation, tachypnea (20 breaths/min), hypertension (195/106 mmHg), and tachycardia (96 bpm). Immediate interventions involved oxygen therapy, cardiac monitoring, ECG, and blood gas analysis, revealing significant acidosis (pH 7.096) and hypoxemia (PO2 78 mmHg). Shortly afterward, ventricular fibrillation occurred, requiring CPR, intubation, and ventilation. Despite initial restoration of sinus rhythm, recurrent cardiac arrests necessitated ongoing resuscitation, vasopressors, and cooling measures. Elevated cardiac biomarkers (cTnT 0.168 ng/mL, NT-proBNP 989 pg./mL), high D-dimer (4.68 μg/mL), and echocardiography confirmed acute massive pulmonary embolism with significant right ventricular strain. During ongoing CPR following the second cardiac arrest, emergency bedside thrombolysis was administered: heparin sodium 3,000 IU intravenous bolus, followed by urokinase 400,000 IU bolus over 3 min and then 600,000 IU infusion over 30 min (total 1,000,000 IU). Following thrombolysis, the patient’s sinus rhythm was temporarily restored but soon deteriorated into cardiac arrest again. The patient was therefore transferred to the Emergency Intensive Care Unit (EICU) for continued management. After stabilization, pulmonary angiography revealed residual thrombus in the left main pulmonary artery. Given the partial response to initial thrombolysis and the availability of interventional radiology services, mechanical thrombectomy was deemed preferable to repeat thrombolysis to minimize bleeding risk. An inferior vena cava filter was placed and mechanical thrombectomy was performed. Subsequent complications, including pleural effusion, rib fractures, and anemia, were effectively managed with thoracic drainage and transfusions. Following comprehensive multidisciplinary care and rehabilitation, the patient steadily improved and was discharged in stable condition several weeks later. Rib fractures were treated conservatively; anemia was corrected with transfusions. No major bleeding was recorded according to ISTH criteria (see Table 1).

**Table 1:** The case comparison.

Parameter	Case 1	Case 2
Age	51	61
Cardiac arrest	Yes	Yes (multiple)
Urokinase dose	1,000,000 units	1,000,000 units
Bleeding complication	None	Rib fracture, anemia
ICU stay	14 days	21 days
Outcome	Full recovery	Full recovery

## 3 Discussion

Successful management of acute fatal pulmonary embolism (PE) demands a well-coordinated, multidisciplinary team approach. Sudden cardiac arrest and hemodynamic instability in these cases require rapid integration of clinical, laboratory, and imaging data to establish the diagnosis promptly. The 2022 ESC guidelines emphasize the importance of a multidisciplinary approach involving a Pulmonary Embolism Response Team (PERT) for managing high-risk PE to facilitate rapid clinical decisions and optimize reperfusion strategies (5). Such teams, comprising specialists from cardiology, interventional radiology, respiratory medicine, and emergency intensive care, enable comprehensive evaluation of thrombus characteristics and guide decisions regarding thrombolysis necessity, timing, and method (6–8). Pulkit et al. have shown that PERT implementation improves survival in high-risk PE cases, aligning with the positive outcomes observed in both patients presented here (4).

Several key clinical features prompted the decision to administer thrombolysis in both cases: (1) profound hypoxemia (SpO₂ < 80%) unresponsive to supplemental oxygen; (2) rapid progression to cardiac arrest within minutes of presentation; (3) echocardiographic evidence of right ventricular strain; and (4) elevated D-dimer levels in the absence of alternative diagnoses. These findings, even without computed tomography pulmonary angiography, provided sufficient justification for emergency thrombolysis.

Treating patients experiencing cardiac arrest from acute PE poses unique challenges due to limited guideline recommendations. However, systemic thrombolysis remains highly effective under these conditions (8). The PEAPETT study supports the initiation of thrombolysis within the first minute of CPR during cardiac arrest due to PE, demonstrating improved return of spontaneous circulation and survival (12).

In the cases described, prompt initiation of intravenous urokinase effectively reduced thrombus burden, restored hemodynamic stability, and facilitated recovery. Urokinase was chosen for its availability in our setting. A bolus of 400,000 units followed by a 600,000-unit infusion was based on institutional protocol. Despite these benefits, thrombolysis carries risks such as bleeding, underscoring the importance of careful individualized risk–benefit evaluation and vigilant post-treatment monitoring (9, 10). The optimal timing, dosage, and thrombolytic administration protocols still require further research (7).

Although generalizability from two cases is limited, rapid diagnosis and early intervention were essential for positive outcomes. Bedside echocardiography, ECG, arterial blood gas analysis, and biomarkers (D-dimer, BNP, cTnT) effectively confirmed acute pulmonary embolism (PE) without relying on pulmonary angiography (11). High-quality cardiopulmonary resuscitation (CPR), despite complications like rib fractures, and targeted temperature management (32–36 °C) were pivotal in achieving favorable neurological outcomes. Prompt intravenous urokinase thrombolysis and structured multidisciplinary coordination via a Pulmonary Embolism Response Team (PERT) significantly stabilized hemodynamic status, while vigilant monitoring and proactive management of complications ensured successful patient recovery.

## 4 Summary

This report emphasizes the critical importance of rapid diagnosis, risk stratification, and timely thrombolysis in managing acute fatal pulmonary embolism. It highlights the value of a multidisciplinary approach via a Pulmonary Embolism Response Team (PERT), ensuring precise thrombolytic administration, effective resuscitation, vigilant monitoring, and proactive complication management. Coordinated, evidence-based care significantly improves survival and recovery outcomes in high-risk PE patients. Although limited by the small sample size and observational design, these cases emphasize the potential life-saving role of rapid thrombolysis and the urgent need for larger-scale trials.

## Data Availability

The original contributions presented in the study are included in the article/supplementary material, further inquiries can be directed to the corresponding author.

## References

[1] Hosokawa Y Yamamoyo T Tanida A Matsuda J Sangen H Nakata J . P3852Twenty-eight-year temporal trends of treatment strategy and short-term outcomes in patients with high-risk pulmonary embolism: impact of ESC guidelines changes. Eur Heart J. (2019) 40:ehz745.0691. doi: 10.1093/eurheartj/ehz745.0691

[2] Kürkciyan I Meron G Sterz F Janata K Laggner AN. Pulmonary embolism as cause of cardiac arrest: presentation and outcome. Arch Intern Med. (2000) 160:1529–35. 10826469 10.1001/archinte.160.10.1529

[3] Yang S-L Gao Y Han Z du X Liu W Jin SG . Successful treatment of near-fatal pulmonary embolism and cardiac arrest in an adult patient with fulminant psittacosis-induced severe acute respiratory distress syndrome after veno-venous extracorporeal membrane oxygenation rescue: a case report and follow-up. Heliyon. (2023) 9:e20562. doi: 10.1016/j.heliyon.2023.e20562, 37842616 PMC10568334

[4] Javaudin F Lascarrou JB Bastard QL Goddet A Bailloul Q Gelin A . Thrombolysis during resuscitation for out-of-hospital cardiac arrest caused by pulmonary embolism increases 30-day survival: findings from the French National Cardiac Arrest Registry. Chest. (2019) 156:1167–75. doi: 10.1016/j.chest.2019.07.015, 31381884

[5] Pruszczyk P Klok FA Kucher N Roik M Meneveau N Sharp ASP . Percutaneous treatment options for acute pulmonary embolism: a clinical consensus statement by the ESC working group on pulmonary circulation and right ventricular function and the European Association of Percutaneous Cardiovascular Interventions. EuroIntervention. (2022) 18:e623–38. doi: 10.4244/EIJ-D-22-00246, 36112184 PMC10241264

[6] Dudzinski DM Piazza G. Multidisciplinary pulmonary embolism response teams. Circulation. (2016) 133:98–103. doi: 10.1161/CIRCULATIONAHA.115. 018337 26719388

[7] Delcroix M de Perrot M Jaïs X Jenkins DP Lang IM Matsubara H . Chronic thromboembolic pulmonary hypertension: realising the potential of multimodal management. Lancet Respir Med. (2023) 11:836–50. doi: 10.1016/S2213-2600(23)00292-8, 37591299

[8] Varshney A Singh JP Vaduganathan M. A heart team approach to contemporary device decision-making in heart failure. Eur J Heart Fail. (2022) 24:562–4. doi: 10.1002/ejhf.2445, 35118780 PMC8986628

[9] Fritze J. "Pulmonary embolism – assessment". In: Springer Reference Medizin. Berlin: Springer. (2023). p. 1–6.

[10] Beydilli İ Yılmaz F Sönmez BM Kozacı N Yılmaz A Toksul İH . Thrombolytic therapy delay is independent predictor of mortality in acute pulmonary embolism at emergency service. Kaohsiung J Med Sci. (2016) 32:572–8. doi: 10.1016/j.kjms.2016.09.004, 27847100 PMC11916484

[11] Thangavel S Korsholm K Veien KT Larsen KM Andersen A. Catheter-directed mechanical thrombectomy in a patient with high-risk pulmonary embolism complicated by out-of-hospital cardiac arrest: a case report. Eur Heart J Case Rep. (2023) 7. doi: 10.1093/ehjcr/ytad307 PMC10358429 37485290

[12] Sharifi M Berger J Beeston P Bay C Vajo Z Javadpoor S. Pulseless electrical activity in pulmonary embolism treated with thrombolysis (from the "PEAPETT" study). Am J Emerg Med. (2016) 34:1963–7. doi: 10.1016/j.ajem.2016.06.094 27422214

